# Childhood Trauma and Sleep Among Young Adults With a History of Depression: A Daily Diary Study

**DOI:** 10.3389/fpsyt.2018.00673

**Published:** 2018-12-04

**Authors:** Jessica L. Hamilton, Ryan C. Brindle, Lauren B. Alloy, Richard T. Liu

**Affiliations:** ^1^Department of Psychiatry, University of Pittsburgh, Pittsburgh, PA, United States; ^2^Department of Psychology and Neuroscience Program, Washington and Lee University, Lexington, VA, United States; ^3^Department of Psychology, Temple University, Philadelphia, PA, United States; ^4^Department of Psychiatry and Human Behavior, Alpert Medical School of Brown University, Providence, RI, United States

**Keywords:** depression, child maltreatment, emotional neglect, insomnia, sleep

## Abstract

Child maltreatment and sleep disturbances are particularly prevalent among individuals with a history of depression. However, the precise relation between child maltreatment and sleep within this population is unclear. The present study evaluated childhood maltreatment and trauma as a predictor of sleep duration and insomnia symptoms among young adults with prior depression. A total of 102 young adults (18–22; 78% female) with a history of clinical or subclinical depression completed an in-person visit with diagnostic interviews and questionnaires of childhood trauma (maltreatment and general trauma), and 2 weeks of daily assessments of sleep and depressive symptoms using internet-capable devices. Using multilevel modeling, we found that only childhood emotional neglect significantly predicted higher levels of insomnia symptoms over the 2 weeks, controlling for daily depression. Neither childhood maltreatment nor trauma predicted sleep duration. Our findings highlight a unique relationship between emotional neglect and insomnia symptoms among individuals with a depression history that, given prior research, may potentially play a role in depression recurrence and represent a potential treatment target.

## Introduction

Childhood trauma is highly prevalent in depression, with over half of individuals with depression endorsing childhood abuse and neglect (1). Meta-analyses indicate that individuals exposed to childhood maltreatment are not only more likely to develop depression, but also experience depression that has a more severe, chronic, and treatment-resistant course [e.g., (1–3)]. Although all types of childhood maltreatment can have lasting consequences, research highlights a distinct relationship between emotional maltreatment and depression, such that emotional abuse and neglect more strongly predict depression course and severity than physical or sexual abuse (1, 3). Given the increased risk of chronic, recurrent depression among those exposed to childhood maltreatment, it is critical to identify potential mechanisms through which childhood maltreatment, particularly emotional abuse and neglect, may confer risk for future depression.

Importantly, sleep disturbance may be one critical mechanism through which individuals exposed to maltreatment are vulnerable for recurrent depressive episodes. Indeed, sleep complaints are among the most common residual symptoms of depression (4, 5). In particular, shorter sleep duration and difficulty initiating and maintaining sleep (i.e., insomnia symptoms) robustly predict depression recurrence (6–8), and are associated with increased risk of suicide (9).

Although research has only recently explored the long-term effects of childhood adversity on sleep in adulthood [for a review see (10)], studies indicate that childhood maltreatment and trauma contributes to poor sleep among adults decades later, even after taking into account intermediate life stress and depression [e.g., (11, 12)]. Further, a recent study of young adults undergoing the college transition prospectively evaluated the impact of specific subtypes of childhood trauma, including emotional, physical, and sexual abuse and neglect, on changes in sleep across the transition (13). This study found that only childhood emotional neglect predicted current psychological distress and subsequent decreases in sleep quality over 6 months later among young adults (13), thereby highlighting a unique relationship between childhood emotional neglect and poor sleep in the context of transitional life stress.

Although childhood maltreatment, and specifically emotional neglect, is associated with both sleep and depression, it remains unclear to what extent childhood trauma contributes to current sleep (duration and insomnia) among clinical populations (e.g., among young adults with a depression history), which may provide valuable information for intervention. Examining the effects of maltreatment and sleep *within* a group of individuals with former depression may yield critical information regarding individual differences in risk, and identify a potential mechanism through which individuals with childhood maltreatment are at heightened risk of recurrent depressive episodes. Given that sleep is a modifiable risk factor, it may represent a potential treatment target for prevention of recurrence among those exposed to child maltreatment and trauma. To date, however, most research has generally relied on retrospective recall of sleep quality over a significant stretch of time (e.g., 6 months), which, in addition to inherent limitations in temporal resolution, could potentially be confounded by current psychological distress.

Thus, as an initial step along this line of empirical inquiry, the present study evaluated the effects of childhood maltreatment on current sleep among young adults with a history of depression. Specifically, we evaluated *both* childhood maltreatment (emotional abuse, emotional neglect, physical abuse, sexual abuse) and general childhood trauma as predictors of sleep duration and insomnia symptoms reported over 2-week daily assessments. Although we hypothesized all forms of childhood maltreatment to be positively associated with insomnia symptoms and shorter sleep duration, we expected a more robust prospective relationship for emotional maltreatment with sleep given prior research indicating a unique role for emotional maltreatment (13). Thus, we expected that emotional abuse and neglect would be the most robust prospective predictors of sleep disturbance (duration, insomnia symptoms), after controlling for current depressive and anxiety symptoms.

## Method

### Participants and Procedure

Participants included young adults (*n* = 102) who participated in the Stress and Emotion study. To participate in the study, participants had to have (1) history of a major or subthreshold depression episode (but not current depression or bipolar disorder), (2) fluency in written and spoken English, and, (3) 18–22 years of age. This sample was selected to ensure sufficient variability of study constructs (i.e., fluctuations in sleep and depressive symptoms) to examine risk for *future* (but not current) depression among those most vulnerable (14). Following a clinical diagnostic interview with trained diagnosticians and informed consent [see (15) for full recruitment procedures], participants completed a baseline assessment with questionnaires about childhood maltreatment, childhood trauma, and an interview about negative life events that occurred in the past 4 weeks. For the next 2 weeks, participants completed daily diary assessments using internet-capable devices (between 6 p.m. and 12 a.m.,) of sleep (sleep/wake times and insomnia symptoms) and current depressive symptoms. This 2-week period was selected to provide a snapshot into participants' sleep and mood, which would provide better representation of individuals' habitual sleep and mood than 1 week without considerably adding participant burden. This study received ethical approval from the Temple University Institutional Review Board.

In the present study, participants on average were 19.86 years old (*SD* = 1.17 years), 78% were female, and 70% self-identified as Caucasian, 15% as African American/biracial, 15% as Asian, and 7% also as Hispanic/Latino. Over 20% identified as lesbian, gay, or bisexual, or “Something else/Other,” and 44% of participants reported maternal education of “some college or less” [used as a proxy for socioeconomic status]. In terms of clinical history, 76 participants (74.5%) met criteria for past major depressive disorder (MDD) and 26 (25.5%) for subthreshold depression, which has a similar course and impairment as MDD (16). The average age of onset of clinical or subclinical depression was 15.62 years (*SD* = 3.31 years).

### Measures

#### Childhood Trauma

To assess childhood trauma, we included two questionnaires that assess specific subtypes of childhood abuse and neglect [Childhood Trauma Questionnaire- CTQ; (17)] and more general childhood traumatic experiences [Trauma History Questionnaire-THQ; (18)].

The CTQ is a 28-item measure that assesses the severity of five subtypes of childhood maltreatment, including emotional abuse (EA), emotional neglect (EN), physical abuse (PA), physical neglect (PN), and sexual abuse (SA). Each subscale consists of five items asking participants to select the extent to which each item is true (“never true” to “very often true”). Higher scores indicate more maltreatment in each domain. The CTQ only examines the extent of maltreatment and does not assess the exact timing or duration. The CTQ has demonstrated excellent psychometric test properties in clinical and non-clinical samples (17, 19).

For more general trauma, participants completed the 24-item Trauma History Questionnaire (THQ), and individuals endorsed the occurrence and age of onset for traumatic events that occurred in their lives in three categories: crime-related (*n* = 4 items), general disaster, and trauma (*n* = 13), and physical and sexual experiences (*n* = 7). Endorsed events are summed in each category. To separately evaluate the impact of more general childhood trauma on sleep, the current study only included events (*n* = 17) in the domains of crime-related (i.e., “Has anyone ever attempted to or succeed in breaking into your home while you were there?”) and general disaster and trauma (i.e., “Have you ever seen someone seriously injured or killed?”) that occurred prior to age 18.

#### Recent Interpersonal Stressors

To account for the effects of recent negative interpersonal events, participants completed the Life Events Scale (LES) and Interview [LEI; (20)], which includes 100 major and minor life events (e.g., school, work, finances, family, peer, and, romantic relationships). Per the gold standard approach for assessing life events (21), participants were interviewed for objective indicators of the occurrence of events to reduce potential biases in event reporting. Events categorized as negative and interpersonal (*n* = 44) were included in the present study. The LES and LEI have demonstrated excellent reliability and validity (20).

#### Anxiety Symptoms

To control for the effects of anxiety on current sleep problems, participants completed the Beck Anxiety Inventory [BAI; (22)]. It is a 21-item measure of anxiety symptoms, and has demonstrated acceptable reliability (22).

#### Daily Depressive Symptoms

In the daily diary, participants reported on daily depressive symptoms using the eight-item PROMIS-Depression-Short Form [SF; (23)], which does not include any sleep items. The PROMIS-Depression-SF also has been found to have sound psychometric properties (23, 24), with α = 0.90 in the present study.

#### Daily Sleep Duration and Insomnia

Also in the daily diary, participants reported the time (hour and minutes) that they went to bed and woke up in the morning, which was used to calculate an estimate of sleep duration. In addition, participants completed three items from the Insomnia Severity Index [ISI; (25, 26)], which assesses the extent of difficulty falling and staying asleep. These items are on a 5-point scale, ranging from 0 (none) to 4 (very severe). The sum of these three items reflected daily insomnia symptoms.

### Statistical Analyses

To better understand the impact of demographic characteristics [race/ethnicity, gender, socioeconomic status (maternal education)] and depression history (clinical vs. subclinical) on experiences of child maltreatment and sleep, we conducted *t-*tests. Given the sample breakdown, race was dichotomized as “Minority” (African American/Black, biracial, Asian, Native American, or Other) and “Non-Minority” (White/Caucasian), and maternal education (proxy for SES) was dichotomized as “Higher” (“College graduate” or more) or “Lower” (“Some college” or lower).

To test the association of childhood trauma with daily-reported insomnia symptoms and sleep duration, we conducted multilevel modeling in Mplus 7.0 (27) with full information maximum likelihood to estimate parameters for missing data. Specifically, our model had two levels of data, including level 1 (within-person) of daily sleep and depressive symptoms and level 2 (between person) of childhood maltreatment and trauma. For analyses, childhood maltreatment subtypes (emotional abuse, neglect, physical abuse, sexual abuse) and general trauma were entered simultaneously as level 2 (between-person) predictors. The outcomes for all analyses were daily sleep parameters of insomnia symptoms and sleep duration [level 1(within-person) variables]. Due to multicollinearity, physical neglect was evaluated separately. For all analyses, we covaried for concurrent daily depressive symptoms (level 1) and between-person (level 2) variables of recent negative interpersonal stressors, anxiety symptoms, and demographic variables (gender, age, race, SES, and prior depression diagnosis).

## Results

Table 1 presents descriptives and bivariate correlations of primary study variables. There were several significant differences of maltreatment by demographic information (gender, race/ethnicity, SES). First, we found that women were more likely to report childhood sexual abuse than men (*t* = 2.33, *p* = 0.01), but there were no other differences on other trauma types or sleep. In addition, minority individuals were more likely to report emotional neglect (*t* = 3.63, *p* < 0.001), physical neglect (*t* = 2.28, *p* = 0.03), and physical abuse (*t* = 4.83, *p* < 0.001) than non-minority individuals. By socioeconomic status (maternal education), there were significant differences for physical abuse (*t* = 3.33, *p* = 0.001) and sexual abuse (*t* = 2.14, *p* = 0.04), and marginal differences in emotional abuse (*t* = 1.75, *p* = 0.08) and emotional neglect (*t* = 1.88, *p* = 0.06). The direction of these relationships was such that individuals with lower socioeconomic status reported more maltreatment. In terms of clinical history, individuals with a major compared to subthreshold depression history were only more likely to experience sexual abuse (*t* = 2.33, *p* = 0.01). There were no other significant differences for other forms of child maltreatment (*t's* < 1.11) or general trauma *(t* = 0.93, *p* = 0.36). Importantly, there were no differences on sleep duration or insomnia symptoms by gender, race/ethnicity, SES, or clinical history.

**Table 1:** Descriptive statistics and bivariate correlations of primary study variables.

**Measure**	**1**	**2**	**3**	**4**	**5**	**6**	**7**	**8**	**9**
1. Childhood EA	–								
2. Childhood EN	0.55	–							
3. Childhood PA	0.21	0.32	–						
4. Childhood PN	0.19	0.53	0.37	–					
5. Childhood SA	0.39	0.19	0.06	0.09	–				
6. General Childhood Trauma	0.04	−0.03	0.01	0.06	0.04	–			
7. Daily Depressive Symptoms	0.15	0.26	0.03	0.20	0.13	−0.03	–		
8. Daily Sleep Duration	−0.01	−0.06	−0.11	0.04	0.04	0.05	−0.23	–	
9. Insomnia	0.24	0.34	0.13	0.06	0.06	−0.02	0.22	−0.15	–
Mean	12.11	9.37	5.79	6.11	5.52	1.08	10.95	7.79	1.59
*SD*	2.99	3.98	1.18	1.79	2.32	1.25	3.10	0.86	1.34

*Correlations > |0.20| are statistically significant (p < 0.05). Mean levels of daily depressive symptoms and sleep are reported. EA, Emotional abuse; EN, Emotional Neglect; PA, Physical Abuse; PN, Physical Neglect; SA, Sexual Abuse*.

### Child Maltreatment and General Trauma With Insomnia Symptoms and Sleep Duration

Consistent with hypotheses for insomnia symptoms, results indicated there was a significant association between childhood emotional neglect with insomnia symptoms over the 2-week period, such that young adults who experienced more childhood emotional neglect reported higher levels of insomnia symptoms over 2 weeks (Table 2). Of note, this was found after covarying for current daily depressive symptoms, recent interpersonal life stress, baseline anxiety symptoms, other forms of childhood abuse and neglect, and demographic characteristics (race, gender, age, SES). Importantly, there were no significant effects of other childhood maltreatment [physical neglect or abuse (emotional, physical, and sexual)] or general childhood trauma.

**Table 2:** Fixed effects of childhood trauma on sleep in young adulthood.

**Variable**	**Insomnia symptoms**			**Sleep duration**		
	***B***	***SE***	***t***	***B***	***SE***	***t***
**BETWEEN-PERSON**						
Intercept	2.63	2.30	1.14	7.38	1.68	4.39^***^
MDD	0.30	0.28	1.07	−0.11	0.20	−0.55
Sex	0.23	0.31	0.75	−0.15	0.23	−0.67
Age	−0.15	0.11	−1.37	0.05	0.08	0.68
Race	−0.32	0.34	−0.94	0.08	0.25	0.33
SES	−0.29	0.26	−1.09	0.13	0.19	0.68
Anxiety	0.01	0.01	0.68	< 0.01	0.01	−0.05
Recent Stress	0.10	0.05	2.11^*^	−0.03	0.04	−0.08
**Child Trauma**						
EA	< −0.01	0.06	−0.09	< 0.01	0.04	0.07
EN	0.10	0.04	2.37^*^	−0.02	0.03	−0.81
PA	0.02	0.12	0.18	−0.06	0.09	−0.66
SA	0.04	0.06	0.62	0.04	0.05	0.88
THQ	−0.07	0.07	−0.97	−0.05	0.05	−0.98
Within–person						
Depression Symptoms	0.006	0.02	0.38	< 0.01	0.02	0.05

* *p < 0.05*;

^**^ *p < 0.01*;

*** *p < 0.001*.

*B represents the unstandardized coefficient. MDD, Major Depressive Disorder (Coded as 0, None; 1, Present); Sex coded as 0, male; 1, female; Race coded as 0, minority; 1, minority; SES coded as 0, lower; 1, higher. EA, Emotional abuse; EN, Emotional Neglect; PA, Physical Abuse; SA, Sexual Abuse; THQ, Trauma History Questionnaire*.

In contrast to our hypotheses, there were no significant effects of childhood maltreatment or trauma on sleep duration (Table 2). This indicates that the effect of childhood emotional neglect was specific to insomnia symptoms, as there were no main effect of emotional neglect on sleep duration in young adults with prior depression.

## Discussion

Childhood trauma can have long-lasting effects on physical and mental health. Although research has consistently linked childhood trauma with depression and, more recently, sleep in adulthood (12, 13), this is the first study to evaluate the relationship between specific subtypes of childhood trauma and sleep in young adulthood among individuals with a depression history. Specifically, we evaluated childhood abuse (emotional, physical, sexual), neglect (physical and emotional), and general childhood trauma (total of crime, environmental, danger) as predictors of insomnia symptoms and sleep duration reported over a 2-week period using a daily diary design.

Overall, our results indicated that childhood emotional neglect uniquely predicted insomnia symptoms among young adults with prior depression, even after accounting for other forms of childhood maltreatment and traumatic experiences, current depressive, and anxiety symptoms, and a range of demographic features that could impact this relationship (gender, race, SES, and depression history). In addition, in contrast to our hypotheses, our results were specific to emotional neglect and insomnia symptoms, as no form of childhood trauma predicted sleep duration. Thus, our results highlight a distinct relationship between emotional neglect during childhood and difficulties initiating and/or maintaining sleep as young adults, which is important given that emotional neglect is one of the most prevalent forms of maltreatment (28). Importantly, given that all individuals had a prior depression history, this is a clinical sample of individuals who are most vulnerable to both childhood trauma exposure and sleep disturbance. This suggests that the effects of emotional neglect on insomnia symptoms may indicate individual differences of future risk for depression recurrence, which is important for identifying sleep as a potential treatment target to improve outcomes and prevent depression recurrence among those with a history of emotional neglect.

Although we hypothesized that emotional neglect would predict both insomnia symptoms and shorter sleep duration, there are several possibilities why childhood emotional neglect may specifically contribute to insomnia symptoms compared to sleep duration and other forms of trauma. Specifically, emotional neglect is a distinct form of maltreatment characterized by the *absence* or *omission* of emotional or psychological support (i.e., child ignored, unresponsive parenting) compared to other types of maltreatment, such as emotional and physical abuse, which are more active acts of *commission*. Further, emotional neglect is described as a pattern of interactions between the caregiver and child rather than specific events or experiences that are *tangible*, such as absence of physical resources (e.g., food). Thus, emotional neglect may uniquely contribute to difficulty in understanding their emotional experiences (29), and impaired biological and cognitive regulation of emotions and perceived stress (30–32). Consequently, emotional neglect may contribute to emotional and social isolation throughout development and into adulthood (31). Given research on the effect of loneliness and both social and emotional isolation on sleep disturbance in adulthood (33, 34), it could be that emotional neglect deprives individuals the emotional safety required for restorative sleep (35), and heighten psychophysiological arousal. Insomnia is characterized by physiological, cognitive, and affective hyperarousal (36); thus, emotional neglect may specifically contribute to insomnia symptoms. Importantly, our study found evidence for the relationship of emotional neglect and insomnia even after taking into account the effect of current interpersonal stressors, which also significantly predict prospective insomnia symptoms. However, it is important for future research to explore the relationship between childhood emotional neglect and current socio-emotional isolation, and empirically evaluate the underlying psychosocial and biological mechanisms linking emotional neglect and sleep disturbance.

Although our study demonstrated the continuing effect of trauma on insomnia symptoms among individuals with depression histories, it is important to note several important directions of future research based on limitations of the current study. For one, it is difficult to disentangle the directionality of these relationships, and it is possible that sleep disturbance preceded the trauma and depression. Given research on the intergenerational transmission of trauma (37) and continuous cycle of abuse (38), it is possible that maltreated individuals have parents who also experienced childhood trauma. Parental experience of trauma could impact neurodevelopmental pathways that contribute to sleep disturbance and depression (39), as well as limited parenting ability to promote sleep hygiene and habits (40). Although we did not assess parental trauma or assess sleep prior to maltreatment exposure, our study did find demographic differences in the experience of emotional neglect by race and socioeconomic status, with minority and lower income individuals reporting more emotional neglect and other forms of maltreatment. However, we did not find any demographic differences in sleep duration or insomnia symptoms. This suggests that despite differences in exposure, reported sleep was less affected by these factors. However, this may indicate individual protective factors that could influence the relationships observed in the study. For instance, it is important to consider to what extent individual and contextual protective factors, such as individual resilience (41), parental sleep monitoring (42), or social support (43), may buffer the effects of maltreatment, which could provide more direct and modifiable targets for intervention.

Our study should be considered in the context of several other limitations, which may influence generalizability and provide critical directions for future research. In particular, this study relied on daily self-reported sleep rather than behaviorally assessed sleep parameters (e.g., actigraphy), which limits our ability objectively to assess sleep duration. Thus, it is possible that emotional neglect (and other forms of maltreatment) may impact sleep duration, but our measure captured *time in bed*, which may not be the most accurate representation of time spent asleep. Further, our study did not assess other sleep domains that could be impacted by child maltreatment (e.g., sleep onset latency, nighttime awakenings). Furthermore, given the high rates of comorbidity of PTSD and depression, particularly among maltreated individuals (44), it is important to recognize that the current study did not assess post-traumatic symptoms or nightmare-related sleep disturbance. It is very possible that individuals with maltreatment histories have post-traumatic symptoms or nightmares related to the trauma that could contribute to the current findings (45). Thus, future research and clinical practice should focus on better understanding whether nightmares and other post-traumatic symptoms, such as hypervigilance, contribute to the relationship observed between childhood emotional neglect and insomnia symptoms in young adulthood.

In addition, we utilized self-report retrospective measures of childhood trauma. Given the length of time that could have elapsed since the trauma, it is possible that individuals may not accurately recall trauma. It is important to note that our study used a prospective daily diary design, which temporally distinguished the timing of childhood maltreatment and sleep assessments. Although this is a strength of the study, it should be noted that it is possible that the 2 weeks captured by the study presents a relatively brief observational period of the association between maltreatment and sleep patterns. Therefore, it is important for future research to carefully consider and incorporate multiple methods and informants to the assessment of maltreatment and sleep. Specifically, though the current study used trauma measures that are widely-used and well-validated (18, 19), clinical interviews would provide more specific information about the maltreatment, including timing, duration, and severity, which may influence the relationships observed in the study. Relatedly, our sample included mostly women and individuals with a history of depression, which limited our ability to test gender differences in these relationships, which is important to consider in future research. In addition, it is unclear the extent to which our findings apply to non-clinical samples (i.e., those without depression) or individuals with later-onset depression. However, given that our sample experienced relatively young first depression onset (15–16 years old), the young adults in this sample may represent a particularly vulnerable group for both sleep disturbance and childhood adversity. Consequently, the fact that we identified emotional neglect as a unique predictor of insomnia symptoms *within* this vulnerable group highlights the importance of examining individual differences within clinical populations.

Importantly, our study provides new insight and preliminary evidence of a distinct relationship between childhood trauma and sleep in young adulthood. Specifically, our findings highlight a unique relationship between childhood emotional neglect and insomnia symptoms among individuals with a history of depression, which may provide important targets of treatment to prevent depression recurrence.

## Author Contributions

JH designed the study, collected the data, and conducted the analyses. JH and RB wrote the initial draft of the manuscript. LA and RL provided substantive revisions to the manuscript.

### Conflict of Interest Statement

The authors declare that the research was conducted in the absence of any commercial or financial relationships that could be construed as a potential conflict of interest.
